# Autophagy contributes to falcarindiol-induced cell death in breast cancer cells with enhanced endoplasmic reticulum stress

**DOI:** 10.1371/journal.pone.0176348

**Published:** 2017-04-25

**Authors:** Tingting Lu, Ming Gu, Yan Zhao, Xinyu Zheng, Chengzhong Xing

**Affiliations:** 1 Department of Anorectal Surgery, the First Hospital of China Medical University, Shenyang, China; 2 Department of Breast Surgery, the First Hospital of China Medical University, Shenyang, China; University of South Alabama Mitchell Cancer Institute, UNITED STATES

## Abstract

Falcarindiol (FAD) is a natural polyyne have been found in many food and dietary plants. It has been found to have various beneficial biological activities. In this study, we demonstrated its anticancer function and mechanism in breast cancer cells. We found that FAD preferentially induces cell death in breast cancer cells. FAD-induced cell death is caspase-dependent. However, FAD induces autophagy to contribute to the cell death. Blocking autophagy by either chemical inhibitors or genetic knockout of autophagy signaling component inhibits FAD-induced cell death. We further found that FAD-induced cell death is mediated by the induction of endoplasmic reticulum stress. We also identified that FAD has synergistic effect with approved cancer drugs 5-FU and Bortezomib in killing breast cancer cells. Summarily, these data demonstrate that FAD has strong and specific anticancer effect in breast cancer cells, and provide some insights about the roles of autophagy in FAD-induced cell death.

## Introduction

Breast cancer is the most frequently diagnosed cancer in women, and ranks second as a cause of cancer death in women. Increasing evidence suggests there are multiple subtypes of breast cancer that occur at different rates and respond to different kinds of treatment. The complexity makes it difficult to control and cure breast cancer [1]. Therefore, novel approaches for the treatment of breast cancer are required. Natural products have been valuable sources of new therapeutic candidate compounds. Identifying new and less toxic natural compounds that can selectively kill cancer cells can potentially lead to the development of better therapy for breast cancer patients [2,3].

Falcarindiol (FAD) is a natural polyyne that have been found in many food and dietary plants [4,5]. FAD has been shown to have anti-inflammation, antibacterial, and anticancer activities, as well as protective effects against hepatotoxicity. These benefits were achieved at non-toxic concentrations and thus represent pharmacologically useful properties [6,7,8,9]. The anticancer activity of FAD was described in some cancer cells [10,11]. Studies found that FAD induced cell death is in colorectal cancer cells, and FAD-induced cell death is related to endoplasmic reticulum (ER) stress [12]. Previous studies showed that ER stress can activate the unfolded protein response (UPR), and induces the splicing of X-box binding protein 1 (XBP1) and increased level of C/EBP-homologous protein (CHOP), and further leads to cell death [12,13,14]. In addition, studies showed that FAD induces autophagy in colorectal cancer cells, but the induced autophagy is not involved in the cell death [12]. So the anticancer function and mechanism of FAD are largely unknown in other cancer cells, and the role of autophagy in FAD-induced cell death is still unclear.

Autophagy is a self-degradative process that is important for balancing sources of energy at critical times in development and in response to nutrient stress. It also plays a housekeeping role in removing misfolded or aggregated proteins. The molecular basis of autophagy has been extensively studied. Autophagy is known to be controlled by a group of autophagy-relative (ATG) genes that control a coordinated process leading to the induction and nucleation of autophagic vesicles, their completion, expansion and fusion with lysosomes and breakdown and recycling. Autophagy is generally thought of as a survival mechanism. However, excessive levels of autophagy have been observed in association with various forms of cell death including apoptosis and necroptosis [15,16]. Accumulated evidences suggest autophagy may be genetically upstream of other death pathways. Knockdown or knockout of ATG genes has been shown to block apoptotic death in many settings, including in murine embryonic fibroblasts that are exposed to ER stress and in p53-overexpessing osteosarcoma cells [17,18]. But the mechanisms that link ATG genes to apoptotic cell death are still largely unknown, it is unclear whether these observations indicate essential roles of the autophagy pathway in triggering apoptosis or rather alternative functions of components of the autophagy machinery in apoptosis signaling or execution [15].

In this study, we demonstrate the anticancer functions of FAD in breast cancer cells. We identified that FAD preferentially kills breast cancer cells. The FAD-induced cell death is caspase-dependent. However, FAD also induces autophagy to contribute to the cell death. Furthermore, we found that FAD-induced cell death is mediated by the induction of ER stress. In addition, we identified that FAD have strong synergistic effect with cancer drugs 5-FU and Bortezomib in killing breast cancer cells. These results suggest that FAD has the potential to be used to development new therapy for breast cancer patients.

## Materials and methods

### Cell culture

MDA-MB-231, MDA-MB-468, SKBR3 and MCF-10A cells were obtained from the American Type Culture Collection (Rockville, MD). MDA-MB-231 and MDA-MB-468 were cultured in DMEM medium supplemented with 10% fetal bovine serum (FBS), 50 IU penicillin/streptomycin, and 2 mmol/l L-glutamine. SKBR3 was cultured in McCoy's 5a medium modified supplemented with 10% FBS, 50 IU penicillin/streptomycin, and 2 mmol/l L-glutamine. MCF-10A were cultured in DMEM/F12 medium supplemented with 5% horse serum, 20 ng/ml EGF, 10 ug/ml Insulin, 0.5 mg/ml Hydrocortisone, 1 mg/ml Cholera Toxin, 50 IU penicillin/streptomycin, and 2 mmol/l L-glutamine from Invitrogen (Carlsbad, CA). All the cells were maintained in a humidified atmosphere with 5% CO_2_ at 37°C.

### Chemicals

FAD was obtained from Haoyuan Chemexpress (Shanghai, China). Z-VAD-fmk was obtained from Promega (Madison, WI). Chloroquine, 3-methyladenine, Necrostatin-1, Cycloheximide, 5-Fluorouracil (5-FU) and proteasome inhibitor PS-341 (bortezomib) were obtained from Sigma (St. Louis, MO).

### Plasmid construction and lentiviral preparation and transduction

Human GRP78 cDNA was sub-cloned into the lentiviral expression vector pCDH-CMVEF1-puro from System Biosciences (Mountain View, CA). The pLKO.1 lentiviral shRNA expression system was used to generate shRNA constructs for GRP78. The sequence of GRP78 shRNA: shGRP78 Oligo1: 5’-CCGGTCTTGTTGGTGGCTCGACTCGACTCGAGTCGAGTCGAGCCACCAACAAGTTTTTT-3’, shGRP78 Oligo2: 5’- AATTAAAAAACTTGTTGGTGGCTCGACTCGACTCGAG TCGAGTCGAGCCACCAACAAGA-3’ [12]. The lentiCRISPRv2 expression system was used to construct lentiviral CRISPR for Atg3 [19]. The sequences of Atg3 CRISPR: Atg3 Oligo1: 5’-CACCGATTACCAGACTCCACGATTA-3’, Atg3 Oligo2: 5’-AAACTAATCGTGGAGTCTGGTAATC-3’. Production of lentivirus was performed as described. Single clone was established after puromycin selection. The genomic DNA of each clone was extracted for PCR to detect the indel mutation in the targeted region. The PCR products were verified by sequencing. Production of lentivirus was performed as described [20].

### Cell viability analysis

Cell viability was assessed by MTT assay. Cells (5 × 10^3^ cells/well) were seeded into 96-well plates. After treatments described in result section, culture medium was replaced with fresh medium containing 0.5 mg/ml MTT from Thermo Fisher Scientific (Waltham, MA), and incubated for 2 h at 37°C. After removing the medium, 100 μl of DMSO was added to each well to solubilize the formazan present in viable cells. The plates were analyzed by measuring the optical density at 540 nm. Experiments were repeated three times.

### FACS analysis of cell death

Cells (1 × 10^5^ cells/well) were seeded into 6-well plates. After indicated treatment, the adherent and detached cells were collected. The cell death was measured by FITC Annexin V Apoptosis Detection Kit from BD Biosciences (San Jose, CA) according the manufacturer’s instructions. The cell mixture was washed twice with cold PBS, and then resuspended at 1×10^6^ cells/mL in binding buffer. Annexin V-FITC and propidium iodide were added to the cell suspension. After gently mixing, the cells were incubated for 15 min at room temperature, and then 400 μl of binding buffer was added to each sample. Quantification of cell death was performed using FACScan flow cytometry from BD Biosciences (San Jose, CA). PI- and/or Annexin V-positive cells were considered as cell death.

### Western blot

Cell lysate was prepared in RIPA buffer (50 mM Tris-HCl pH8.0, 150 mM NaCl, 0.1% SDS, and 0.5% Na deoxycholate, 1% NP40) with fresh proteinase inhibitor cocktail from Roche (Basel, Switzerland). Samples were quantified by Bradford reagent from Sigma (St. Louis, MO), and measured at 595 nm with a microplate reader. Equal amount of protein (10 μg) was loaded. After standard transfer and block, the sample membrane was incubated with primary antibody for overnight at 4°C. After standard wash, the membrane was incubated with secondary antibody Goat anti-mouse/Rabbit IRDye from Li-Cor. IRDye fluorescent dyes have absorption and emission wavelengths in the near-infrared spectrum, between 680 and 800 nm. The western signals can be detected and analyzed using a Li-Cor Odyssey image reader by software Image Studio (Ver. 2.1) from Li-Cor (Lincoln, NE). Antibodies used: LC3A/B (4108, dilution 1:1000), Atg3 (3415, dilution 1:1000), and GRP78 (3177, dilution 1:1000) from Cell Signaling (Danvers, MA), β-Actin (AC-15, dilution 1:3000) from Santa Cruz (Dallas, TX), Goat anti-mouse/Rabbit IRDye (dilution 1:10000) from Li-Cor (Lincoln, NE).

### RNA Isolation and RT-PCR

Total RNA for RT-PCR was isolated from cells using TRIzol from Invitrogen (Carlsbad, CA). Total RNA (2 μg) was reverse-transcribed using M-MLV reverse transcriptase from Promega (Madison, WI) and random primers following manufacturer’s protocol. PCR was performed in triplicate using FastStart PCR Master reagents from Roche (Basel, Switzerland) in PCR System from Bio-rad (Hercules, CA) under the following conditions: 3 min at 95°C followed by 30 cycles of 95°C for 20 s 60°C for 40 s and 72°C for 1 min. PCR products were analyzed by standard agarose gel electrophoresis. The PCR products were loaded onto agarose gel containing ethidium bromide from Sigma (St. Louis, MO). After finishing the gel electrophoresis, the gel was visualized by using UV light. A photo was taken. Primers used: XBP1 forward: 5’-GGAGTTAAGACAGCGCTTGG-3’, XBP1 reverse: 5’-ACTGGGTCCAAGTTGTCCAG-3’; CHOP forward: 5’-TGGAAGCCTGGTATGAGGAC-3’, CHOP reverse: 5’-TGTGACCTCTGCTGGTTCTG-3’; GAPDH forward: 5’-CTCTGACTTCAACAGCGACAC-3’, GAPDH reverse: 5’- CATACCAGGAAATGAGCTTGACAA -3’.

### Statistical analysis

The data were collected from at least three independent experiments. Values are presented as mean ± S.D. Statistical significance was assessed by Student’s two-tailed *t*-test and P<0.05 was considered statistically significant.

## Results

### FAD preferentially induces cell death in breast cancer cells

**Fig 1:**
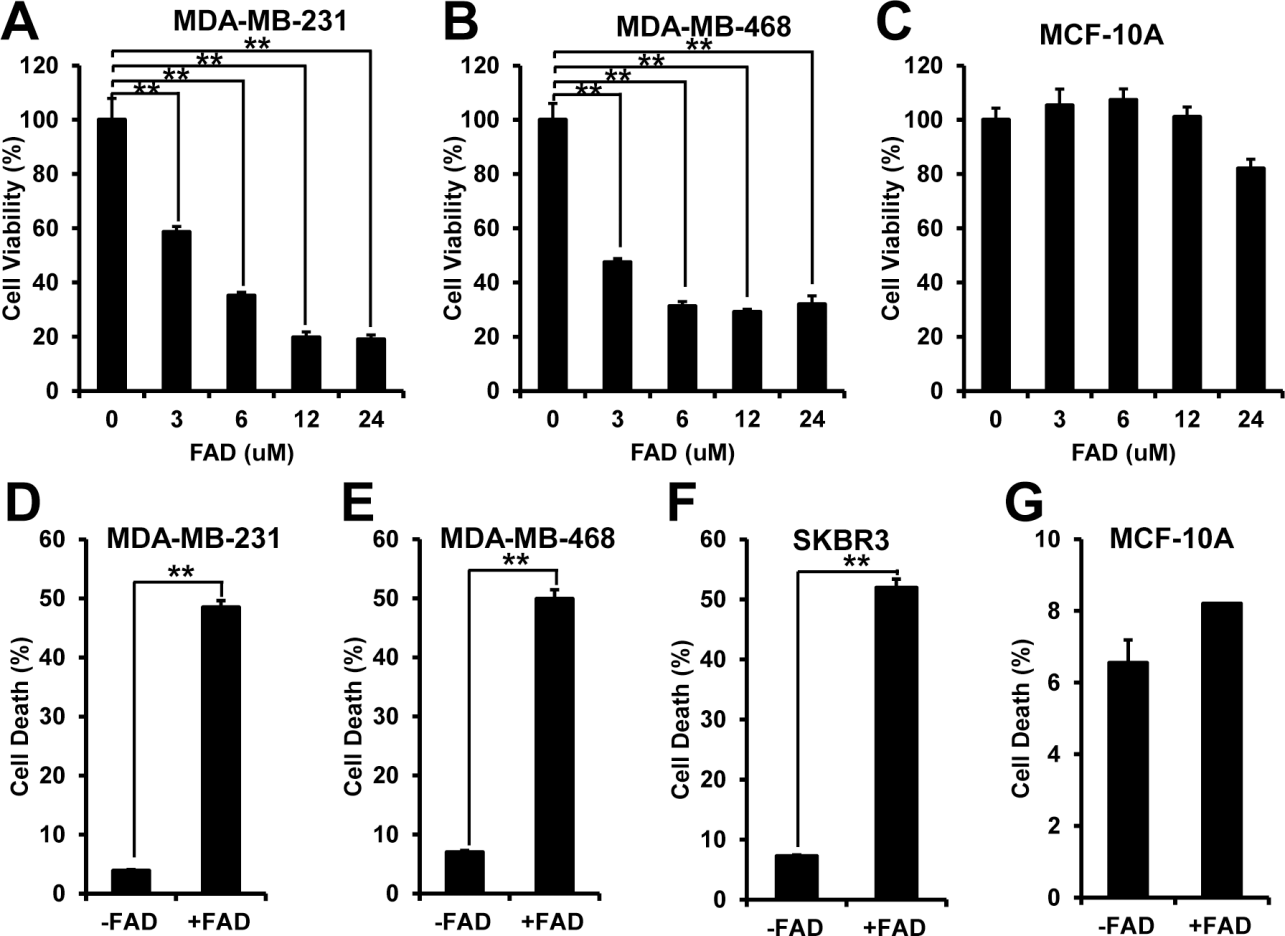
FAD preferentially induced cell death in breast cancer cells. (A-C) Cell viability of breast cancer cell MDA-MB-231 (A), MDA-MB-468 (B) and breast normal cell MCF-10A (C) was measured by MTT assay in the presence of FAD in the indicated concentrations; (D-G) Cell death of breast cancer cell MDA-MB-231 (D), MDA-MB-468 (E), SKBR3 (F) and breast normal cell MCF-10A (G) was measured by Annexin V and PI assay with or without FAD. The data were collected from at least three independent experiments. Values are presented as mean ± S.D. **p<0.01.

To determine the effect of FAD on breast cancer cells, we first compared cell viability between breast cancer cells MDA-MB-231, MDA-MB-468 and normal breast cells MCF-10A after FAD treatment. The cells were treated with the indicated doses of FAD for 24 hours, and then the cell viability was measured by MTT assay. The data showed that cell viability of MDA-MB-231 and MDA-MB-468 cells was significantly decreased in the presence of FAD. About 60% of cell viability was lost at 6 uM of FAD (Fig 1A and 1B). However, cell viability of MCF-10A cells was unchanged until the dose of FAD reached to 24 uM (Fig 1C). We next measured cell death by Annexin V and PI assay. Both MDA-MB-231 and MDA-MB-468 cells showed significantly increased cell death after FAD treatment (Fig 1D and 1E).

MDA-MB-231 and MDA-MB-468 cells are triple negative breast cancer cells, which have no expression of estrogen receptor (ER), progesterone receptor (PR) and no amplification of HER-2/Neu. We want to test whether FAD has effect on other subtypes of breast cancer cells. SKBR3 is a human breast cancer cell line that has no expression of ER and PR, but has amplification of Her2 gene [21]. We found that FAD also induced significant cell death in SKBR3 cells (Fig 1F). In the same condition, no significant increase of cell death was found in MCF-10A cells (Fig 1G). Summarily, FAD preferentially induces cell death in breast cancer cells.

### Autophagy contributes to FAD-induced cell death in breast cancer cells

**Fig 2:**
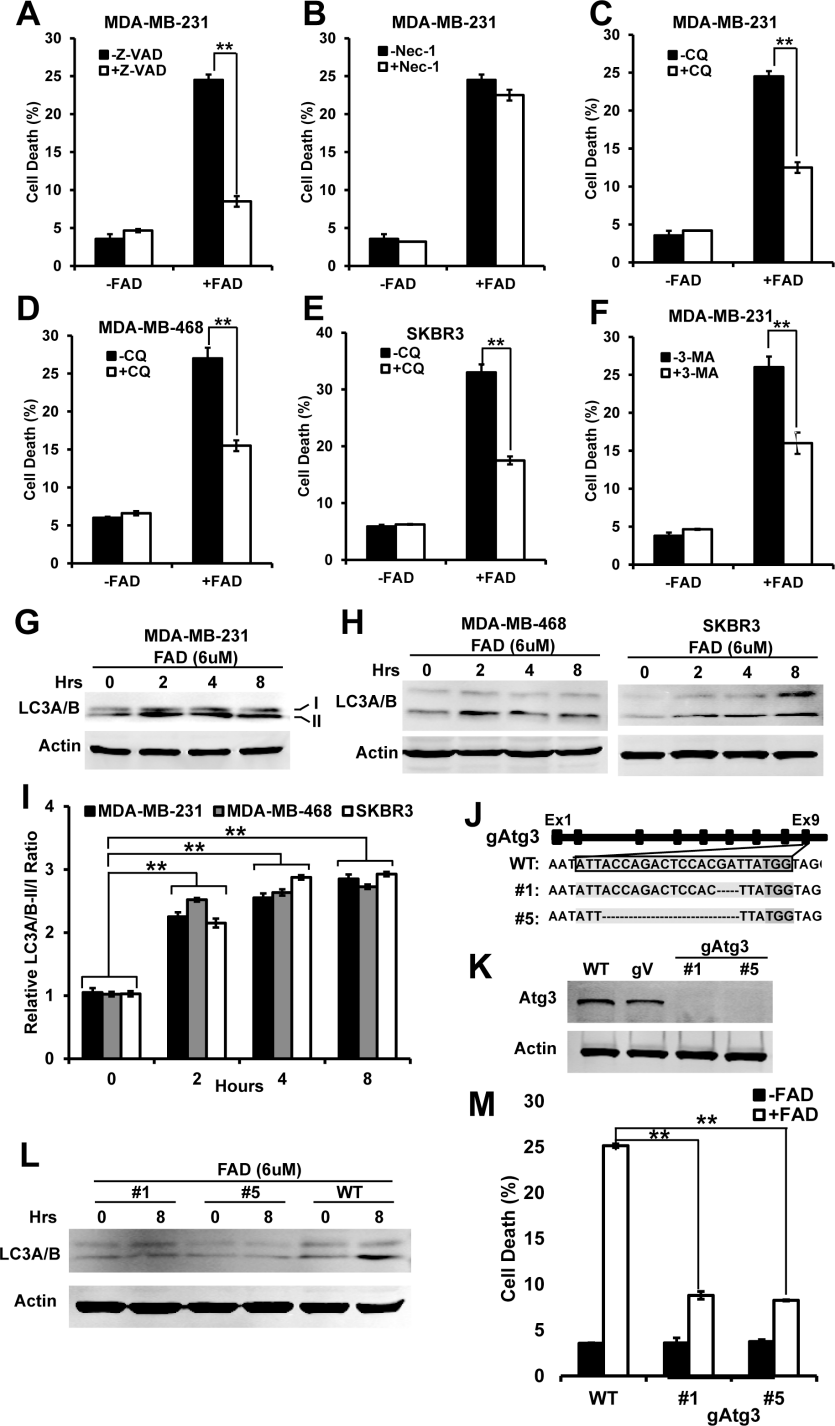
Autophagy contributes to FAD-induced cell death in breast cancer cells. (A) Cell death of MDA-MB-231 cells treated with 6 uM of FAD with or without 100 uM of Z-VAD-fmk (Z-VAD); (B) Cell death of MDA-MB-231 cells treated with 6 uM of FAD with or without 40 uM of Necrostatin-1 (Nec-1); (C-E) Cell death of MDA-MB-231 (C), MDA-MB-468 (D) and SKBR3 (E) cells treated with 6 uM of FAD with or without 20 uM of chloroquine (CQ); (F) Cell death of MDA-MB-231 cells treated with 6 uM of FAD with or without 5 mM of 3-methyladenine (3-MA); (G) and (H) The western blot analyzed the level of LC3A/B-II/I in the presence of 6 uM FAD for indicated hours in MDA-MB-231 (G), MDA-MB-468 and SKBR3 (H) cells. The level of β-Actin was served as a loading control; (I) The calculated relative ratio of LC3A/B-II/I in breast cancer cells basing on band intensity. The data were collected from at least three independent experiments; (J) Sequence analysis of indel mutations in targeted regions of Atg3 locus in MDA-MB-231 cell clones. sgRNA sequences is shown in light gray shadow and the PAM sequence is in dark gray shadow. Deleted sequence is shown in dashed line; (K) The western blot analyzed the level of ATG3 in single Atg3 CRISPR clones with wild type (WT) and empty vector targeted (gV) cells as controls; (L) The western blot analyzed the level of LC3A/B-II/I in the presence of 6 uM FAD for indicated hours in Atg3 CRISPR clones. The level of β-Actin was served as a loading control; (M) Cell death of MDA-MB-231 wild type cells and Atg3 CRISPR clones was measured by Annexin V and PI assay with or without FAD. The data were collected from at least three independent experiments. Values are presented as mean ± S.D. **p<0.01.

Three types of cell death are usually happened in cancer cells: caspase-dependent apoptosis, necrosis and autophagic cell death. So we like to know which kind of cell death that induced by FAD in breast cancer cells. In above cell death assay, we used Annexin V to detect apoptotic cells, and see a significant increase in Annexin V stained cells. It suggests that FAD induces significant level of apoptosis. To support this, we treated cells with pan caspase inhibitor Z-VAD-fmk in the presence of FAD. We found that Z-VAD significantly inhibited the cell death induced by FAD (Fig 2A). Recent studies show that necroptosis, a programmed form of necrosis, is specifically inhibited by necrostatins [22]. So we used a necroptosis inhibitor Necrostatin-1 to test whether necrosis plays roles in FAD-induced cell death. We found that Necrostatin-1 does not inhibit FAD-induced cell death (Fig 2B). It indicates necrosis is not included in FAD-induced cell death.

To study whether autophagy is involved in FAD-induced cell death, we used chloroquine, an autophagy inhibitor, to block autophagy to study the effect in cells. We found that blocking autophagy by chloroquine significantly decreased the FAD-induced cell death in MDA-MB-231, MDA-MB-468 and SKBR3 cells (Fig 2C–2E). We then used another autophagy inhibitor 3-methyladenine (3-MA) to confirm the above data. The 3-MA was the first identified and is the most widely used autophagy inhibitor [23]. We found that 3-MA significantly decreased the FAD-induced cell death in MDA-MB-231 (Fig 2F). These data suggest that the autophagy contributes to the cell death.

We next want to know whether FAD induces autophagy in breast cancer cells. Upton autophagy induction, the microtubule-associated light chain 3 (LC3-I) is cleaved to form the faster migrating LC3-II form, which is conjugated to phospholipids and incorporated into the autophagosomes. The conversion of LC3-I to LC3-II is often used as markers for autophagy induction [24]. So we examined the effect of FAD on the induction of LC3-II. We found that significant level of LC3-I was converted to LC3-II after the cells were treated with for 6 uM of FAD for 2 hours in MDA-MB-231, MDA-MB-468 and SKBR3 cells (Fig 2G–2I). It indicates that FAD induces autophagy.

To further confirm the roles of autophagy in FAD-induced cell death, we genetically blocked autophagy pathway by targeting autophagocytosis associated protein ATG3 using CRISPR knockout system. ATG3 is the E2 enzyme for the LC3 lipidation process. It is essential for autophagocytosis, and known to play a role in regulation of autophagy during cell death [25,26]. The sequencing results and the immune blot data showed the correct knockout of ATG3 in the indicated CRISPR single clones (Fig 2J and 2K). We further confirmed that the level of LC3-II in gAtg3 cells was unchanged under FAD treatment (Fig 2L). It indicates that FAD-induced autophagy response was destructed in these cells. With these cells, we found that FAD-induced cell death was significantly rescued (Fig 2M). It suggests that autophagy contribute to FAD-induced cell death.

### ER stress mediates FAD-induced cell death in breast cancer cells

**Fig 3:**
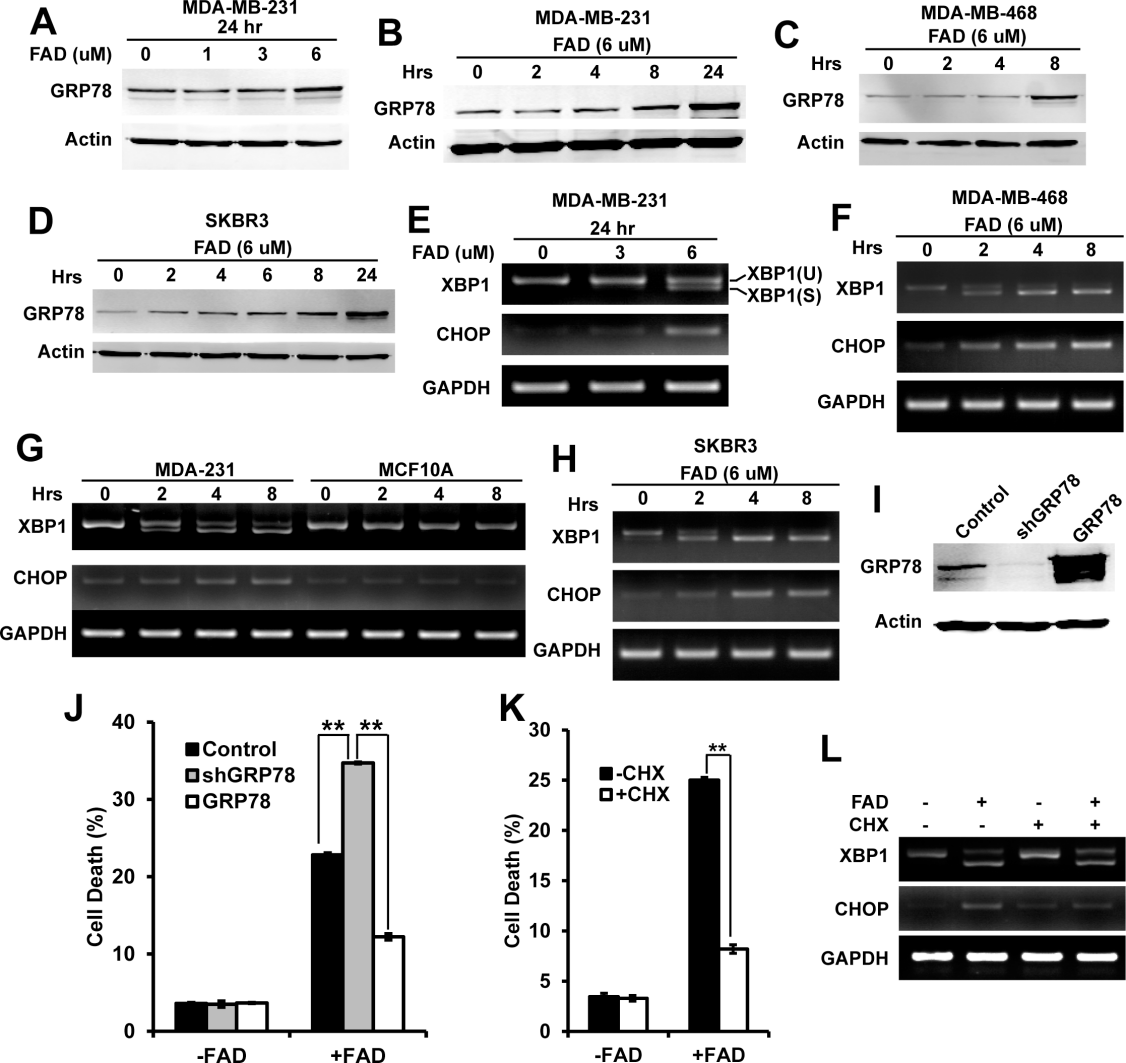
FAD-induced cell death in breast cancer cells is mediates by ER stress. (A) The western blot analyzed the level of GRP78 in MDA-MB-231 cells treated with indicated concentration of FAD for 24 hours; (B-D) The western blot analyzed the level of GRP78 in MDA-MB-231(B), MDA-MB-468 (C) and SKBR3 (D) cells treated with 6 uM of FAD for indicated time; (E) RT-PCR analyzed the level of splicing XBP1 and CHOP in MDA-MB-231 cells treated with indicated concentration of FAD for 24 hours; (F) RT-PCR analyzed the level of splicing XBP1 and CHOP in MDA-MB-468 cells treated with 6 uM of FAD for indicated time; (G) RT-PCR analyzed the level of splicing XBP1 and CHOP in MDA-MB-231 and MCF10A cells treated with 6 uM of FAD for indicated time; (H) RT-PCR analyzed the level of splicing XBP1 and CHOP in SKBR3 cells treated with 6 uM of FAD for indicated time; (I) The western blot analyzed the level of GRP78 in MDA-MB-231 knockdown and GRP78 overexpression cells; (J) Cell death of MDA-MB-231 shGRP78 knockdown and GRP78 overexpression cells was measured by Annexin V and PI assay with or without FAD; (K) Cell death of MDA-MB-231 cells treated with 6 uM of FAD with or without 10 ug/ml of cycloheximide (CHX); (L) RT-PCR analyzed the level of splicing XBP1 and CHOP in MDA-MB-231 cells treated with or without 6 uM of FAD in the presence or absence of CHX. The data were collected from at least three independent experiments. Values are presented as mean ± S.D. **p<0.01.

FAD-induced cell death in colorectal cancer cells is caused by increased ER stress. To test whether FAD induces ER stress in breast cancer cells, we first studied whether FAD induce the expression of GRP78. GRP78 is an ER chaperone that functions as an inhibitor of ER stress receptors and inhibits ER stress-mediated cell death [27]. We found that FAD increased the level of GRP78 in MDA-MB-231 cells in dose- and time-dependent manner (Fig 3A and 3B). We also found that FAD increased the level of GRP78 in MDA-MB-468 and SKBR3 cells (Fig 3C and 3D). At 6 uM of FAD, the level of GRP78 was induced in cells after 8–24 hours of treatment. We next tested whether the expression of CHOP and splicing of XBP1 were induced by FAD. The RT-PCR results showed the increased level of CHOP and splicing of XBP1 in both dose- and time-dependent manner (Fig 3E and 3G). We also identified that FAD induced expression of CHOP and splicing of XBP1 in MDA-MB-468 and SKBR3 cells (Fig 3F and 3H). As early as 2 hours, FAD induced expression of CHOP and splicing of XBP1 were detected, significantly earlier than the induction of GRP78. Significant changes in the level of CHOP and splicing of XBP1 were only found in breast cancer cells, and not found in normal MCF-10A cells (Fig 3G). Summarily, the specifically FAD-induced ER stress in cancer cells suggests the ability of FAD to induce ER stress correlates with FAD-induced cell death.

The above data suggest that FAD-induced cell death is mediated by ER stress. To test this idea, we manipulated ER stress by overexpression or knockdown of GRP78 to measure the effect on FAD-induced cell death. The immunoblot data showed the expected down-regulation of GRP78 by shRNA knockdown and up-regulation of GRP78 by overexpression (Fig 3I). The cell death assay showed that knockdown of GRP78 significantly increased FAD-induced cell death, and overexpression of GRP78 significantly decreased FAD-induced cell death (Fig 3J). It indicates that FAD-induced cell death is mediated by ER stress.

FAD-induced ER stress could be caused by the intracellular accumulation of proteins [12]. To characterize it in breast cancer cells, we determined the effect of blocking protein synthesis by cycloheximide (CHX) on FAD-induced cell death. The data showed that CHX significantly rescued the FAD-induced cell death (Fig 3K). To study whether CHX rescued FAD-induced cell death through mediating ER stress, we test the level of CHOP and splicing of XBP1 in cells after CHX treatment. The RT-PCR data showed decreased level of CHOP and accumulated unsplicing form of XBP1 in CHX and FAD treated cells (Fig 3L). It indicates that FAD increases ER stress by inducing protein accumulation.

### FAD has synergistic effect with 5-FU and Bortezomib in killing breast cancer cells

**Fig 4:**
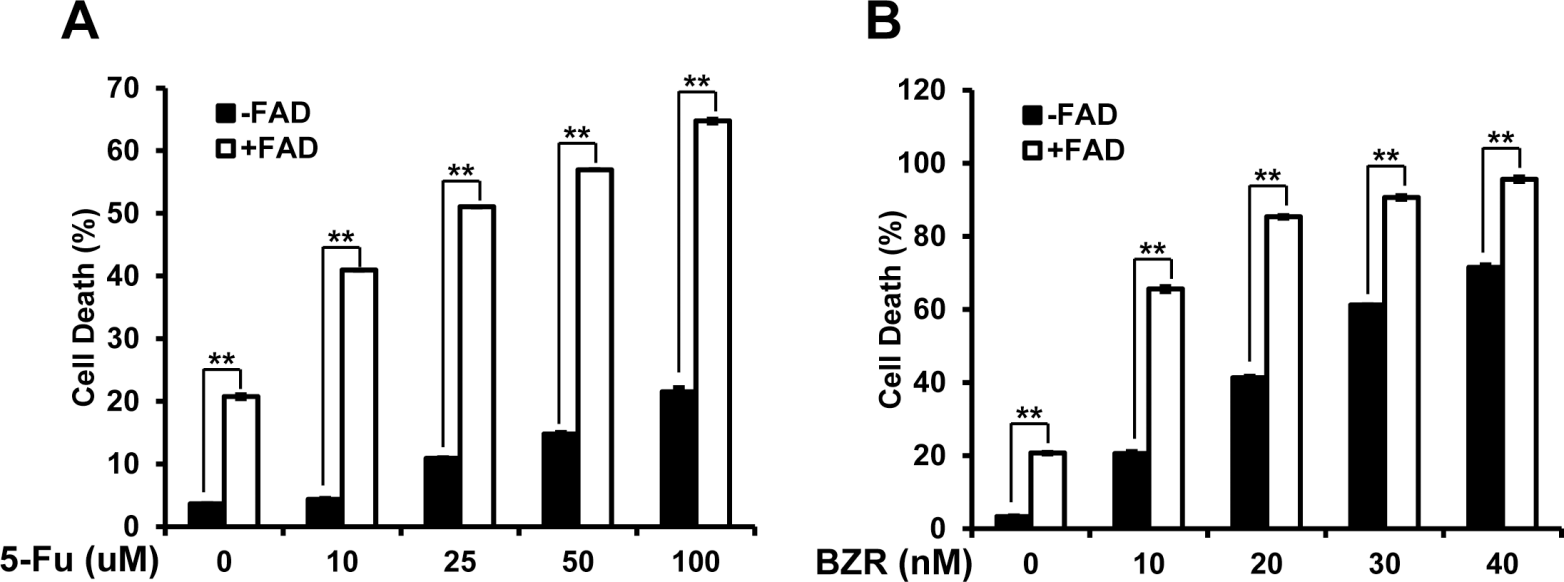
FAD shows synergistic effect with 5-FU and Bortezomib in killing breast cancer cells. (A) Cell death of MDA-MB-231 cells treated with 0, 10, 25, 50 and 100 uM of 5-FU was measured by Annexin V and PI assay with or without 1 uM of FAD; (B) Cell death of MDA-MB-231 cells treated with 0, 10, 20, 30 and 40 nM of Bortezomib (BZR) was measured by Annexin V and PI assay with or without 1 uM of FAD. The data were collected from at least three independent experiments. Values are presented as mean ± S.D. **p<0.01.

Considering the well-accepted concept of combination cancer therapy, we are interested in testing whether FAD can be used in combination with currently approved therapeutic drugs to enhance its ability in killing cancer cells and reduce the toxicity to normal cells. We first study FAD combination effect with 5-FU. 5-FU is a chemotherapy drug to treat different cancers. We treated MDA-MB-231 cells with the indicated amount of 5-FU with or without the presence of 1 uM of FAD. We found that, in the presence of 1 uM of FAD, 5-FU showed a significantly stronger cytotoxic effect on MDA-MB-231 cells at much lower concentrations (Fig 4A). It suggests that FAD enhanced 5-FU ability in killing breast cancer cells.

We next test FAD combination effect with Bortezomib (Velcade). Borezomib is the first proteasome inhibitor clinically approved for treating relapsed multiple myeloma and mantle cell lymphoma [28]. Studies also reported its ability in killing breast cancer cells [29]. We treated MDA-MB-231 cells with the indicated amount of Borezomib with or without the presence of 1 uM of FAD. In the presence of 1 uM of FAD, Borezomib showed a significantly stronger cytotoxic effect on MDA-MB-231 cells at much lower concentrations (Fig 4B). It indicates FAD and Bortezomib induce strong synergistic cell death effect. In summary, the above data support that FAD has a strong synergistic effect with both 5-FU and Bortezomib in killing breast cancer cells, so it can potentially be used to increase efficiency of these approved drugs, and decrease the dose and side effect of the drugs.

## Discussion

FAD was found in many food and dietary plants with many benefits. The anticancer effect of FAD has recently been reported. Jin et al. reported that FAD induces cell death in colorectal cancer cells, and also suggested that the similar effect and mechanisms in several cancer cells derived from different tissues [12]. In our study, we further demonstrated the anticancer effects and related mechanisms of FAD in multiple breast cancer cell lines in details. We found the FAD preferentially induces cell death in two subtypes of breast cancer cells, including triple negative cancer cells (MDA-MB-231 and MDA-MB-468) and Her2 positive cancer cells (SKBR3). More interestingly, we first identified the role of autophagy in FAD-induced cell death. We also provided solid evidence to support that ER stress mediates FAD-induced cell death in multiple breast cancer cells. Furthermore, we revealed that FAD has a strong synergistic effect with cancer drugs 5-FU and Bortezomib in killing breast cancer cells, and can potentially limit the side effect from these drugs. In summary, these data demonstrate the anticancer effects of FAD and related mechanisms in breast cancer cells, and suggests that FAD has the potential to lead to new drug for breast cancer treatment.

In the study, we provide several lines of evidences to support the role of autophagy in FAD-induced cell death in breast cancer cells. The evidences include the induction of autophagy after FAD treatment in multiple breast cancer cell lines, the inhibited FAD-induced cell death after the destruction of autophagy by two autophagy chemical inhibitors, and inhibited FAD-induced cell death after disrupting autophagy signaling through knockout of autophagy component Atg3. This observation is different to previous findings in colorectal cancer cells. As mentioned earlier, people showed that FAD-induced autophagy does not contribute to cell death in colorectal cancer cells [12]. But it is not surprising to find that autophagy is involved in the FAD-induced cell death in breast cancer cells. Studies showed that autophagy might function upstream of other death pathway, such as apoptosis [15]. Espert et al. showed that siRNA against two ATG genes completely suppressed apoptosis in T lymphocytes [30]. Furthermore, previous studies found some autophagy proteins can also function directly as a pro-apoptotic factor to initiate apoptosis. Calpain-mediated cleavage of Atg5 switches autophagy to apoptosis [31]. Disrupting Atg12-Atg3 complex formation produces an expansion in mitochondrial mass and inhibits cell death mediated by mitochondrial pathways [32]. However, to understanding the detail mechanism of autophagy in FAD-induced cell death requires further studies.

Our data suggest that FAD has a strong synergistic effect with chemotherapy drug 5-FU and proteasome inhibitor bortezomib. The synergistic effect may be due to either stress sensitization or overload in the combinations. Cancer cells generally exhibit increased cellular stresses, and more require stress support pathway for survival. This makes cancer cells more susceptible to either stress sensitization or overload than normal cells [33]. Previous studies have suggested that stress sensitization or overload is likely to be the mechanism of chemotherapy drugs. Our data show that FAD kills breast cancer cells through inducing ER stress. Agents that target distinct or same branches of cellular stress response potentially lead to synergistically in inducing stress overload in cancer cells. Consistent with that, FAD and 5-FU, which induces ER stress and DNA damage stress respectively, show synergistic effects in killing cancer cells. Several studies have suggested that Bortezomib induces ER stress through interfering with ERAD, and further contributes to its cytotoxic activity against cancer cells [34]. FAD and Bortezomib, which both induces ER stress, also exhibit synergistic effects in killing cancer cells.

## Conclusions

Our studies demonstrated the specific anticancer effect of FAD in breast cancer cells. We revealed that FAD-induces autophagy plays an important part in cell death caused by enhanced ER stress. We identified that FAD showed strong synergistic effects with approved anti-cancer drugs 5-FU and Bortezomib. FAD may be a potential novel therapeutic agent for the treatment of human breast cancer.
